# Improving Lipid Screening Adherence in Inflammatory Arthritis Using a Two-Cycle Plan-Do-Study-Act (PDSA) Quality Improvement Intervention

**DOI:** 10.7759/cureus.113930

**Published:** 2026-08-04

**Authors:** Morgan Lilly, Mahnoor Javed, Deepak Jagannath, Kanika Monga

**Affiliations:** 1 School of Engineering Medicine, Texas A&amp;M University Naresh K. Vashisht College of Medicine, Houston, USA; 2 Rheumatology, Houston Methodist Hospital, Houston, USA

**Keywords:** ankylosing spondylarthritis, lipid screening, plan-do-study-act (pdsa), preventative, quality improvement projects, rheumatoid arthritis, rheumatology, risk factors cardiovascular diseases, spondyloarthritis

## Abstract

Introduction: Autoimmune diseases, such as rheumatoid arthritis (RA) and spondylarthritis (SpA), including ankylosing spondylitis (AS) and psoriatic arthritis (PsA), are characterized by chronic systemic inflammation and are associated with significant cardiovascular morbidity and mortality. Dyslipidemia is a well-established contributor to this increased cardiovascular risk and represents an important comorbidity in patients with rheumatologic disease.

Study aim: This pre-post interventional quality improvement (QI) study used two iterative Plan-Do-Study-Act (PDSA) cycles at a high-volume, academic rheumatology clinic to improve provider adherence to lipid screening guidelines for patients with seropositive RA, PsA, or AS, as determined by International Classification of Diseases 10th Revision (ICD-10) codes. Patients were excluded if they had a diagnosis of systemic lupus erythematosus (SLE), juvenile dermatomyositis, Sjogren syndrome, or arthropathic psoriasis, or if they were managed at other rheumatology clinics within the Houston Methodist system.

Methods: Baseline screening rates were established via retrospective chart review between July 2024 and February 2025. PDSA Cycle 1 spanned from March to April of 2025 and involved the deployment of provider education, physical and email reminders, and a custom Epic dot phrase. PDSA Cycle 2 spanned from April to May of 2025 and consisted of pre-visit chart review and the placement of a pending lipid panel into the electronic health record (EHR) if needed. The primary outcome was the percentage of eligible patients with a completed lipid panel in the preceding 12 months.

Results: Retrospective chart review at baseline indicated a care gap, with 62.4% (98/157) of eligible patients having completed a lipid panel within the prior 12 months. Interventions for PDSA Cycle 1 resulted in the rheumatology providers ordering two lipid panels for patients, yet the total screening rate with the intervention was 57.1% (28/49), lower than baseline. The process interventions in PDSA Cycle 2 drastically increased the lipid screening rate to 92.8% (13/14).

Conclusions: The QI initiative demonstrated that system-level workflow interventions incorporating pre-visit chart review and preemptive order entry were associated with improved lipid screening adherence among patients with RA, PsA, and AS. Educational interventions and passive EHR-based tools alone did not result in sustained improvement. These findings highlight the importance of embedding preventive care measures directly into clinical workflows to improve adherence to cardiovascular risk screening guidelines in rheumatology practice.

## Introduction

Autoimmune diseases, such as rheumatoid arthritis (RA) and spondyloarthritis (SpA), including ankylosing spondylitis (AS) and psoriatic arthritis (PsA), are characterized by chronic systemic inflammation and are associated with significant cardiovascular morbidity and mortality. Dyslipidemia is a well-established contributor to this increased cardiovascular risk and represents an important comorbidity in patients with rheumatologic disease [1]. As a result, understanding the relationship between autoimmune disease and dyslipidemia is a growing area of clinical concern.

Patients with RA, for instance, comprise 0.5-1% of the population and have a 48% higher risk of cardiovascular disease (CVD) than the general population, and the European Alliance of Associations for Rheumatology (EULAR) guidelines recommend CVD screening in these patients annually [2]. Similarly, patients with SpA have significant associations with dyslipidemia, hypertension, and other adverse cardiovascular comorbidities across a variety of retrospective and cross-sectional studies [3-5].

The pathogenesis of dyslipidemia in rheumatologic disease is primarily driven by systemic inflammation and proinflammatory cytokines. For instance, in RA, increased levels of IL-6, IL-1, and TNF-α alter lipid metabolism, promoting low-density lipoprotein (LDL) oxidation and accelerating atherosclerosis [6]. This is also seen in SpA, and further studies have revealed varied contributors to dyslipidemia, including the involvement of the PCSK9 pathway [7]. Interestingly, the proinflammatory state seen in patients with RA has been associated with decreased total cholesterol and levels of high-density lipoprotein (HDL) and LDL. This paradoxical association does not correlate with decreased CV risk; instead, it suggests that lower lipid levels should be targeted in these patients compared to the general population [8]. Overall, the data and literature to date support routine screening for dyslipidemia and aggressive risk factor modification in patients with autoimmune disease.

Based on the strong association between dyslipidemia and autoimmune diseases, as well as the subsequent complications due to CVD, numerous guidelines have been established regarding the screening of these high-risk patients. The American Heart Association (AHA) recommends that patients with RA or SpA be lipid screened at least annually. This is supported by the EULAR guidelines, which also advocate for annual CV risk assessments for patients with RA and a strong consideration for patients with AS and PsA [9]. The US Preventive Services Task Force (USPTF) takes a broader perspective, recommending lipid screening at least every five years in patients with autoimmune disease, or every two years with CV risk factors [10].

This was previously presented as a poster abstract at the 2025 Academic College of Rheumatology Convergence Meeting in October 2025.

## Materials and methods

Context

This was a single-clinic, pre-post interventional quality improvement (QI) study conducted at the Academic Houston Methodist Rheumatology Clinic in the Texas Medical Center. The initiative intended to improve provider adherence to lipid screening guidelines for patients with RA, PsA, and AS. The clinic is high volume, with each physician seeing approximately 18 patients per day. 

The site utilizes the Epic electronic health record (EHR) system. Epic is a widely used EHR system in the healthcare industry that supports clinical decision-making, documentation, and ordering of treatments. Customizations can be performed for an organization or an individual, such as the creation and development of documentation templates or a dot phrase. A dot phrase acts like a keyboard shortcut that populates a specific template, phrase, or text field after typing ".XXX," where Xs represent the name of the dot phrase to load.

Patient population and eligibility

Study participants included rheumatology fellows and attending physicians managing applicable patient encounters. Eligible encounters were identified via ICD-10 codes for RA, PsA, and AS. The ICD-10 code category for each disease appears in Table 1, while the comprehensive list of ICD codes included in the study appears in Appendix 1. Patients were excluded if they had a diagnosis of systemic lupus erythematosus (SLE), juvenile dermatomyositis, Sjogren syndrome, arthropathic psoriasis, or if they were managed at other rheumatology clinics within the Houston Methodist system.

**Table 1:** Categories of ICD-10 codes included in this study for RA, PsA, and AS ICD-10: International Classification of Diseases 10th Revision; AS: ankylosing spondylitis; PsA: psoriatic arthritis; RA: rheumatoid arthritis

Disease	ICD-10 Code
Seropositive Rheumatoid Arthritis	M05, M05.7-M05.9
Psoriatic Spondyloarthritis	L40, L40.5-L40.9
Ankylosing Spondylitis	M45, M45.0-M45.AB

Ethical considerations

The project was reviewed by the Houston Methodist Institutional Review Board and determined to be exempt.

QI framework

**Figure 1:**
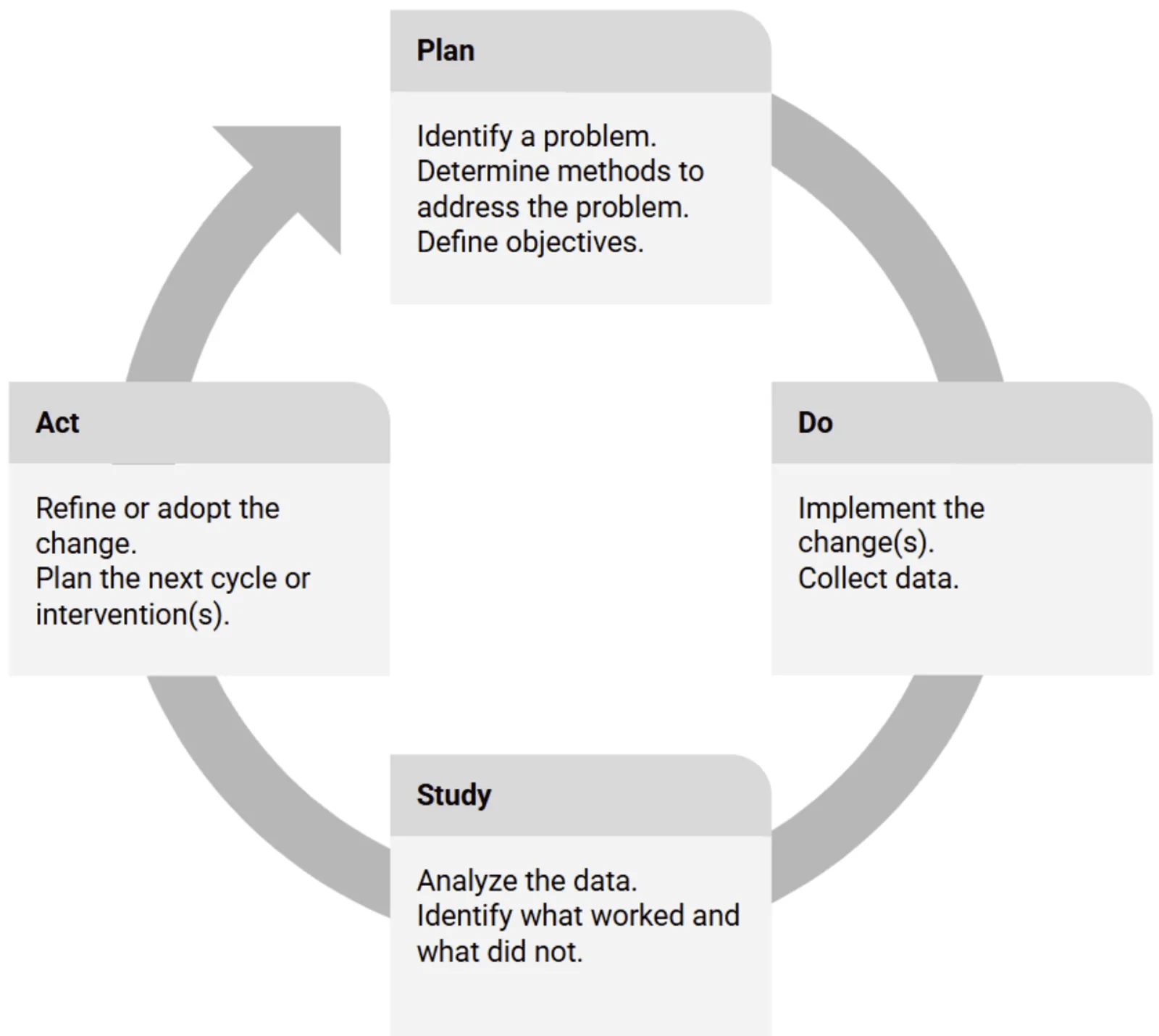
Plan-Do-Study-Act quality improvement cycle Image created by the Medical Team in Microsoft PowerPoint (Microsoft® Corp., Redmond, WA)

The study followed the Plan-Do-Study-Act (PDSA) cycle methodology for QI. PDSA is a structured QI tool shown to facilitate iterative improvement by setting aims, defining measurements, implementing changes, testing interventions in a real-world clinical setting, and reflecting on outcomes to guide refinement, as shown in Figure 1 [11,12]. Two PDSA cycles were executed sequentially. 

PDSA Cycle 1 

Plan

To establish baseline performance and confirm a care gap between clinical practice and recommended lipid screening guidelines, a retrospective chart review was performed. Eligible encounters within the academic rheumatology clinic were identified in the EHR between July 2024 and February 2025 using the ICD-10 inclusion criteria previously described. 

Do

Following the baseline data analysis, the first intervention cycle was implemented from March to April 2025 and spanned eight weeks. At the beginning of this study period, the study team provided an educational session for providers highlighting the increased cardiovascular risk in patients with autoimmune diseases and reviewing current lipid screening guidelines. Concurrently, a custom Epic dot phrase was deployed to facilitate rapid documentation of LDL levels and provide a visual reminder to order lipid panels when necessary. Physical workstation reminders and emails were distributed to providers within the clinic to reinforce compliance.

Study

After the Cycle 1 intervention period, the team retrospectively reviewed individual charts of patients seen meeting the inclusion criteria between March and April of 2025. The primary outcome was defined as the percentage of eligible patients with a completed lipid panel in the preceding twelve months, including panels ordered by rheumatology providers during the encounter. 

Act

Limited improvement in screening adherence prompted the team to implement a process-level intervention in a second PDSA cycle. 

PDSA Cycle 2 

Plan

The team planned an active, process-driven intervention that incorporated the review of patient charts prior to the visit, thereby minimizing cognitive burden. 

Do

Over a four week-period spanning April and May of 2025, the team screened eligible patients prior to clinic visits for a lipid panel completed within the prior twelve months. For patients out of compliance, a pending lipid panel was entered into Epic for rheumatology providers to review and sign during the encounter.

Study

The team again reviewed individual patient charts for eligible encounters during this study period to measure the primary outcome of lipid screening compliance within the past year. 

Act

Due to a substantial increase in guideline adherence achieved during this PDSA cycle, the clinic implemented pre-visit charting and order-pending into the clinic's standard workflow.

## Results

Baseline (July 2024-February 2025)

A total of 157 patients met the inclusion criteria during the baseline period. Of these, 98 patients (62.4%) had completed a lipid panel screening within the past 12 months, confirming an initial care gap of 37.6% (59/157). 

PDSA Cycle 1 (March-April 2025)

During the first cycle spanning eight weeks, 49 eligible patients with RA, AS, or PsA were identified. Among this cohort, 26 patients (53.1%) had a documented lipid panel within the prior 12 months. Of the remaining 23 patients (46.9%) lacking recent testing, fellows placed new lipid panel orders for two patients (8.7% of the sub-cohort). After accounting for these new orders, the overall screening adherence rate during Cycle 1 was 57.1% (28/49), representing a marginal decrease relative to baseline. 

PDSA Cycle 2 (April-May 2025)

In the second interventional cycle spanning four weeks, 14 patients were identified as lacking a recent lipid panel prior to their scheduled encounter. The implementation of this active, process-level workflow resulted in a substantial increase in guideline adherence, with 13 of the 14 patients (92.8%) receiving appropriate lipid screening.

A comprehensive summary of the longitudinal lipid screening adherence rates across all study phases is detailed in Table 2.

**Table 2:** Lipid screening rates across baseline and Plan-Do-Study-Act (PDSA) cycles

Phase	Total Eligible Patients (N)	Patients Already Compliant at Visit % (n/N)	Non-compliant Patients Targeted (n)	New Lipid Panels Ordered (n)	Overall Screening Adherence % (n/N)
Baseline	157	62.4% (98/157)	–	–	62.4% (98/157)
PDSA Cycle 1	49	53.1% (26/49)	23	2	57.1% (28/49)
PDSA Cycle 2	14	0% (0/0)	14	13	92.8% (13/14)

## Discussion

This QI initiative addressed a critical care gap in lipid screening adherence for high-risk rheumatology patients through process interventions. Characterized by chronic systemic inflammation, autoimmune diseases are associated with significant cardiovascular morbidity and mortality. As a result, national guidelines, including those from the American College of Cardiology (ACC) and the American College of Rheumatology (ACR), recommend routine lipid testing for these populations to enable accurate calculation of the atherosclerotic cardiovascular disease (ASCVD) risk score and to guide therapy in high-risk patients [13,14]. Despite these guidelines, retrospective chart review confirmed the presence of a gap, with only 62.4% (98/157) of eligible patients meeting screening recommendations within the academic rheumatology clinic.

Initial efforts during PDSA Cycle 1 focused on provider education, the implementation of a dot phrase in the EHR, and passive reminders. These interventions resulted in a lower screening rate of 57.1% (28/49) compared to baseline. It is noteworthy that two lipid panels were ordered during PDSA Cycle 1, attributable to the education and reminders provided by the system.

Akenroye et al. similarly demonstrated that passive electronic health record-based reminders alone were insufficient to meaningfully improve cardiovascular risk screening in RA populations [15]. This highlights the limited effectiveness of provider-dependent interventions. In that study, despite integration of automated reminders within the clinical workflow, screening rates remained largely unchanged, suggesting that alert fatigue and competing cognitive demands may limit the impact of passive strategies implemented in an EHR. These findings are consistent with the present study, in which education and reminder-based interventions alone did not produce meaningful improvement in adherence. This aligns with the hierarchy of intervention effectiveness, which conceptualizes education, reminders, and checklists as lower-tier strategies that rely heavily on individual behavior [16]. On the contrary, system-level design changes, such as forcing functions and workflow redesign, are more effective at producing sustained improvement. In contrast, more structured, team-based approaches involving workflow redesign and interprofessional engagement have been shown to produce more sustained improvements in task additions in clinical practice.

In contrast, the second PDSA cycle involved process modifications, such as pre-visit chart review and lipid panel pending. This system-level modification resulted in a 92.8% (13/14) compliance rate, a significant improvement from baseline. These findings are consistent with Bartels et al., who demonstrated that rheumatology clinic protocols incorporating structured interprofessional workflows improve population-level cardiovascular risk management, including follow-up and blood pressure control [17].

The findings of this study should also be interpreted in the context of existing QI literature in rheumatology. Published PDSA and QI initiatives within rheumatology remain limited and highly heterogeneous. Prior studies have primarily focused on disease activity monitoring, depression screening, and medication safety, with fewer addressing cardiovascular risk screening. These trends have been summarized in integrative reviews of rheumatology QI initiatives, which highlight variability in intervention design, outcomes, and implementation approaches across studies [18].

For example, Bays et al. performed two PDSA cycles that focused on interventions to improve documentation and tracking of the clinical disease activity index in RA patients [19]. Similar to our QI project, the researchers saw limited improvement or changes in PDSA Cycle 1 after implementation of a tool requiring physicians to remember to complete it. In their study, they added a form for a physician to complete, while our team added optional dot phrases in Epic. Furthermore, both studies implemented workflow changes in the second PDSA cycle that involved pre-chart review and identification of eligible patients for the interventions, and both resulted in significant improvements in adherence to the respective interventions. Similarly, Mulvihill et al. demonstrated that workflow modifications involving pre-visit planning were more effective in improving adherence to depression screening in childhood-onset systemic lupus erythematosus compared to education alone [20]. Cintron et al. also reported comparable findings in a lupus QI initiative focused on mycophenolate safety monitoring [21]. This study also involved iterative PDSA cycles that ultimately improved adherence to pregnancy screening and risk mitigation strategies for patients on mycophenolate by incorporating new structured workflow processes into the clinical environment.

While these studies support the feasibility of QI methodologies in rheumatology, they differ substantially in clinical focus, intervention design, and outcome measures, limiting direct comparability to lipid screening initiatives. However, they collectively reinforce that workflow-integrated interventions are more effective than passive, provider-dependent strategies across rheumatology QI contexts.

Taken together, the limited number of directly comparable rheumatology QI studies highlights a broader gap in structured, iterative PDSA-based interventions targeting cardiovascular risk screening. Within this constrained literature base, our findings align with prior work suggesting that system-level, workflow-integrated interventions are more effective than passive or education-based strategies for improving preventive care delivery in specialty clinics. Collectively, these findings suggest that, while provider education and EHR-based reminders may contribute modestly to awareness, they are insufficient to drive sustained improvements in preventive care delivery without concurrent workflow integration.

Future work

Future work will focus on linking screening to management, coordinating care with cardiology or primary care providers (PCP), as well as monitoring longitudinal outcomes. To further optimize the rheumatology workflow, lipid panel results may be auto-populated directly into the standard rheumatology clinic template note. This could streamline documentation, decrease cognitive burden during patient encounters and pre-chart review, and reduce duplicate data entry.

A collaborative approach between rheumatologists and cardiologists or PCPs may be essential to ensure that patients with autoimmune diseases are appropriately screened for dyslipidemia and initiated on statin therapy if needed. To support this, the team is considering providing a targeted educational session to cardiologists and PCPs that emphasizes the elevated cardiovascular risks and recommended screening guidelines for patients with autoimmune diseases. While recognizing that education alone can have a limited yield, it may be a good place to initiate collaboration between groups. Concurrent integration of process-level interventions like pre-charting reviews and pending lipid panels with these partnering physicians may be explored thereafter.

Finally, the team may investigate the average ASCVD risk among the patient population studied. If a high baseline risk exists across this population, the team will investigate the utility of embedding an automated ASCVD risk assessment calculator into the standard rheumatology clinic template. This could provide real-time risk stratification that further guides cardiovascular management of impacted patients. 

Limitations

This single-center QI study in an academic rheumatology clinic may limit generalizability, and reliance on a single EHR system (Epic) may reduce applicability to other settings. Screening was evaluated at the encounter level and may be subject to the Hawthorne effect, due to provider awareness of the initiative. The follow-up period was relatively short, and intervention phases differed in duration due to real-world clinic and trainee turnover. Consistent with PDSA-based QI methodology, no formal hypothesis testing was performed. Despite these limitations, substantial improvements in lipid screening adherence support the effectiveness of the implemented interventions.

## Conclusions

Patients with autoimmune diseases, such as RA, PsA, and AS, are at higher risk for cardiovascular complications and therefore require more frequent lipid screening than the general population. This QI initiative identified a significant care gap, with only 62.4% of patients meeting the screening recommendations at baseline. In this QI initiative, provider education, reminders, and optional EHR tools resulted in minimal improvement, while implementation of a structured pre-visit review process with pending lipid panel orders substantially increased adherence. These findings are consistent with prior rheumatology literature and suggest that system-level workflow modifications are more effective than passive, provider-dependent interventions.

The success of this initiative provides a practical and scalable framework for incorporating cardiovascular risk screening into routine rheumatology practice. Future efforts should focus on linking screening to management; strengthening interdisciplinary collaboration among rheumatology, cardiology, and primary care; and evaluating the long-term impact of these interventions on cardiovascular risk assessment and treatment.

## Appendices

Appendix 1: ICD-10 codes

**Table 3:** Comprehensive list of ICD-10 codes included in the study population Source: Ref [22]

M05	Rheumatoid arthritis with rheumatoid factor
M05.7	Rheumatoid arthritis with rheumatoid factor without organ or systems involvement
M05.70	Rheumatoid arthritis with rheumatoid factor of unspecified site without organ or systems involvement
M05.71	Rheumatoid arthritis with rheumatoid factor of shoulder without organ or systems involvement
M05.711	Rheumatoid arthritis with rheumatoid factor of right shoulder without organ or systems involvement
M05.712	Rheumatoid arthritis with rheumatoid factor of left shoulder without organ or systems involvement
M05.719	Rheumatoid arthritis with rheumatoid factor of unspecified shoulder without organ or systems involvement
M05.72	Rheumatoid arthritis with rheumatoid factor of elbow without organ or systems involvement
M05.721	Rheumatoid arthritis with rheumatoid factor of right elbow without organ or systems involvement
M05.722	Rheumatoid arthritis with rheumatoid factor of left elbow without organ or systems involvement
M05.729	Rheumatoid arthritis with rheumatoid factor of unspecified elbow without organ or systems involvement
M05.73	Rheumatoid arthritis with rheumatoid factor of wrist without organ or systems involvement
M05.731	Rheumatoid arthritis with rheumatoid factor of right wrist without organ or systems involvement
M05.732	Rheumatoid arthritis with rheumatoid factor of left wrist without organ or systems involvement
M05.739	Rheumatoid arthritis with rheumatoid factor of unspecified wrist without organ or systems involvement
M05.74	Rheumatoid arthritis with rheumatoid factor of hand without organ or systems involvement
M05.741	Rheumatoid arthritis with rheumatoid factor of right hand without organ or systems involvement
M05.742	Rheumatoid arthritis with rheumatoid factor of left hand without organ or systems involvement
M05.749	Rheumatoid arthritis with rheumatoid factor of unspecified hand without organ or systems involvement
M05.75	Rheumatoid arthritis with rheumatoid factor of hip without organ or systems involvement
M05.751	Rheumatoid arthritis with rheumatoid factor of right hip without organ or systems involvement
M05.752	Rheumatoid arthritis with rheumatoid factor of left hip without organ or systems involvement
M05.759	Rheumatoid arthritis with rheumatoid factor of unspecified hip without organ or systems involvement
M05.76	Rheumatoid arthritis with rheumatoid factor of knee without organ or systems involvement
M05.761	Rheumatoid arthritis with rheumatoid factor of right knee without organ or systems involvement
M05.762	Rheumatoid arthritis with rheumatoid factor of left knee without organ or systems involvement
M05.769	Rheumatoid arthritis with rheumatoid factor of unspecified knee without organ or systems involvement
M05.77	Rheumatoid arthritis with rheumatoid factor of ankle and foot without organ or systems involvement
M05.771	Rheumatoid arthritis with rheumatoid factor of right ankle and foot without organ or systems involvement
M05.772	Rheumatoid arthritis with rheumatoid factor of left ankle and foot without organ or systems involvement
M05.779	Rheumatoid arthritis with rheumatoid factor of unspecified ankle and foot without organ or systems involvement
M05.79	Rheumatoid arthritis with rheumatoid factor of multiple sites without organ or systems involvement
M05.7A	Rheumatoid arthritis with rheumatoid factor of other specified site without organ or systems involvement
M05.8	Other rheumatoid arthritis with rheumatoid factor
M05.80	…… of unspecified site
M05.81	Other rheumatoid arthritis with rheumatoid factor of shoulder
M05.811	Other rheumatoid arthritis with rheumatoid factor of right shoulder
M05.812	Other rheumatoid arthritis with rheumatoid factor of left shoulder
M05.819	Other rheumatoid arthritis with rheumatoid factor of unspecified shoulder
M05.82	Other rheumatoid arthritis with rheumatoid factor of elbow
M05.821	Other rheumatoid arthritis with rheumatoid factor of right elbow
M05.822	Other rheumatoid arthritis with rheumatoid factor of left elbow
M05.829	Other rheumatoid arthritis with rheumatoid factor of unspecified elbow
M05.83	Other rheumatoid arthritis with rheumatoid factor of wrist
M05.831	Other rheumatoid arthritis with rheumatoid factor of right wrist
M05.832	Other rheumatoid arthritis with rheumatoid factor of left wrist
M05.839	Other rheumatoid arthritis with rheumatoid factor of unspecified wrist
M05.84	Other rheumatoid arthritis with rheumatoid factor of hand
M05.841	Other rheumatoid arthritis with rheumatoid factor of right hand
M05.842	Other rheumatoid arthritis with rheumatoid factor of left hand
M05.849	Other rheumatoid arthritis with rheumatoid factor of unspecified hand
M05.85	Other rheumatoid arthritis with rheumatoid factor of hip
M05.851	Other rheumatoid arthritis with rheumatoid factor of right hip
M05.852	Other rheumatoid arthritis with rheumatoid factor of left hip
M05.859	Other rheumatoid arthritis with rheumatoid factor of unspecified hip
M05.86	Other rheumatoid arthritis with rheumatoid factor of knee
M05.861	Other rheumatoid arthritis with rheumatoid factor of right knee
M05.862	Other rheumatoid arthritis with rheumatoid factor of left knee
M05.869	Other rheumatoid arthritis with rheumatoid factor of unspecified knee
M05.87	Other rheumatoid arthritis with rheumatoid factor of ankle and foot
M05.871	Other rheumatoid arthritis with rheumatoid factor of right ankle and foot
M05.872	Other rheumatoid arthritis with rheumatoid factor of left ankle and foot
M05.879	Other rheumatoid arthritis with rheumatoid factor of unspecified ankle and foot
M05.89	…… of multiple sites
M05.8A	…… of other specified site
M05.9	Rheumatoid arthritis with rheumatoid factor, unspecified
M45	Ankylosing spondylitis
M45.0	Ankylosing spondylitis of multiple sites in spine
M45.1	Ankylosing spondylitis of occipito atlanto axial region
M45.2	Ankylosing spondylitis of cervical region
M45.3	Ankylosing spondylitis of cervicothoracic region
M45.4	Ankylosing spondylitis of thoracic region
M45.5	Ankylosing spondylitis of thoracolumbar region
M45.6	Ankylosing spondylitis lumbar region
M45.7	Ankylosing spondylitis of lumbosacral region
M45.8	Ankylosing spondylitis sacral and sacrococcygeal region
M45.9	Ankylosing spondylitis of unspecified sites in spine
M45.A	Non radiographic axial spondyloarthritis
M45.A0	…… of unspecified sites in spine
M45.A1	…… of occipito atlanto axial region
M45.A2	…… of cervical region
M45.A3	…… of cervicothoracic region
M45.A4	…… of thoracic region
M45.A5	…… of thoracolumbar region
M45.A6	…… of lumbar region
M45.A7	…… of lumbosacral region
M45.A8	…… of sacral and sacrococcygeal region
M45.AB	…… of multiple sites in spine
L40.5	Arthropathic psoriasis
L40.50	…… unspecified
L40.51	Distal interphalangeal psoriatic arthropathy
L40.52	Psoriatic arthritis mutilans
L40.53	Psoriatic spondylitis
L40.54	Psoriatic juvenile arthropathy
L40.59	Other psoriatic arthropathy
L40.8	Other psoriasis
L40.9	Psoriasis, unspecified
